# Safety Pin Suture for Management of Atonic Postpartum Hemorrhage

**DOI:** 10.5402/2012/405795

**Published:** 2012-04-05

**Authors:** Ali Abdelhamed M. Mostfa, Mostafa M. Zaitoun

**Affiliations:** Department of Obstetrics and Gynecology, Faculty of Medicine, Zagazig Universiy, P.O. Box 44519, Sharqia, Egypt

## Abstract

*Objective*. To assess the efficacy of a new suture technique in controlling severe resistant uterine atonic postpartum hemorrhage. *Patients and Methods*. This is a retrospective observational study that included thirteen women with uterine atony and postpartum bleeding that did not react to usual medical management. All these women underwent compressing vertical suture technique in which the anterior and posterior walls of the uterus were attached so as to compress the uterus. The suture is transfixed at the uterine fundus, thus eliminating the risk of sutures sliding off at the uterine fundus (safety pin suture). *Results*. safety pin uterine compression suture was a sufficient procedure to stop the bleeding immediately in 92.2% of the women. None of the women developed complications related to the procedure. *Conclusion*. A new safety pin suture is a simple and effective procedure to control bleeding in patients with treatment-resistant, life-threatening atonic postpartum hemorrhage with the advantage of eliminating the risk of the sutures sliding off at the uterine fundus.

## 1. Introduction

Primary postpartum hemorrhage (PPH) is a major obstetric complication that can follow delivery leading to catastrophic event (hysterectomy and/or maternal death) in patients not responding to medical treatment. Even in a saved patients it may be a major cause of maternal morbidity such as renal failure and/or Sheehan's syndrome (a potentially life-threatening complication).The most common cause of PPH is uterine atony, which is responsible for at least 80 percent of cases [1]. Although risk factors and preventive measures are clearly known, some cases of PPH are unexpected. In developing countries, home vaginal deliveries (which still present in many areas) are poorly managed with increased risk of labor abnormalities that represent a risk factor to PPH and sometimes unavoidable hysterectomy. The general management of atonic PPH starts by conservative measures (uterine massage and uterotonics drugs) escalating to unavoidable surgical interventions (internal iliac artery ligation and hysterectomy) to control severe life-threatening bleeding [2]. Surgical uterine compression sutures, a mechanical method of compressing the uterus and closing the arterial bed to reduce bleeding, have been developed to avoid emergency hysterectomy and to preserve fertility in these patients [3–6].

## 2. Patients and Method

This is a retrospective observational study carried out at Obstetric and Gynecology Departments, Zagazig University Hospitals, Egypt on, 13 patients presented by severe atonic PPH in the period from April 2008 to December 2010. In all 13 patient, routine initial measures (uterine massage, oxytocin infusion, methergine, if not contraindicated, and misopristol) were done and when failed, compressing uterine suture was performed.

### 2.1. Description of the Safety Pin Suturing Technique

(1) Under general anesthesia, the patient is catheterized and placed in the Lloyd Davies position for easy access to the vagina to assess the control of bleeding.

(2) The abdomen is opened by a Pfannenstiel incision or if the patient had caesarean section following which she bled, the same incision is reopened.

(3) The uterus is exteriorised and rechecked to identify any bleeding point, if no obvious bleeding point is observed then bimanual compression is first tried to assess the potential efficacy of the compression suturing technique. The vagina is swabbed out to confirm adequate control of bleeding.

(4) If vaginal bleeding is controlled, the procedure is as follows.

**Figure 1:**
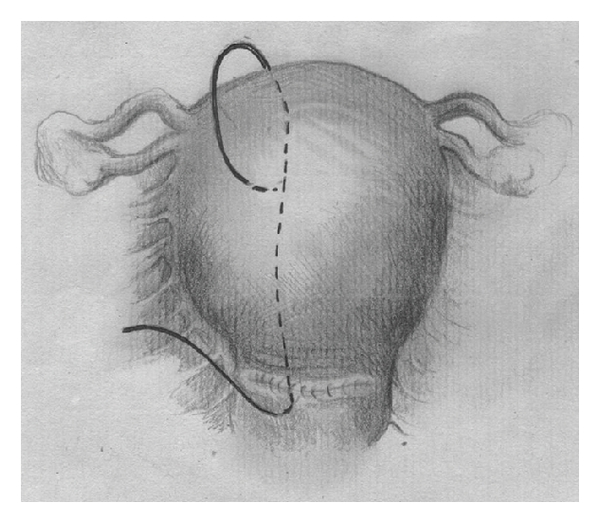


**Figure 2:**
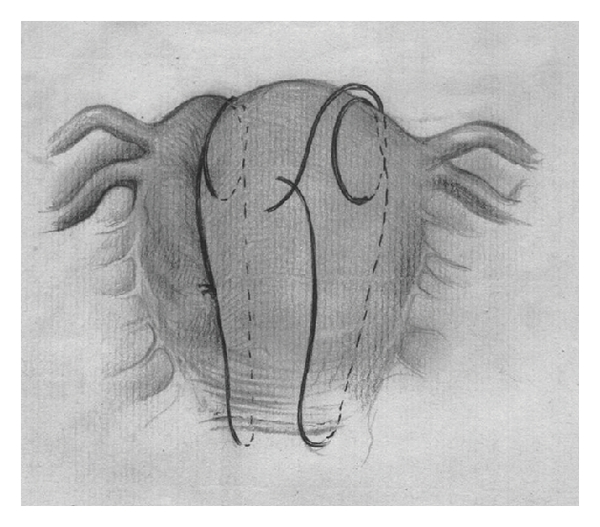


**Figure 3:**
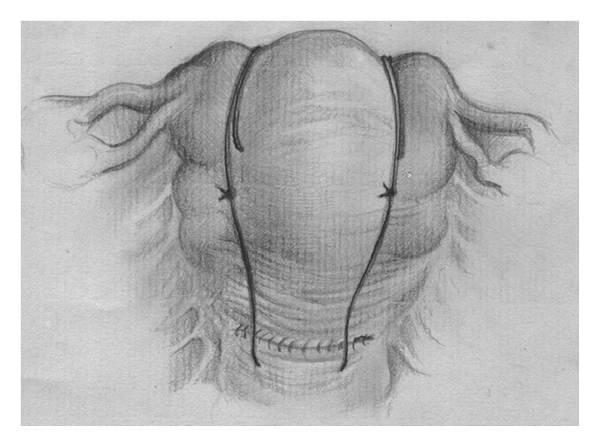


 Catgut 2, 70 mm round needle (Demetech corporation, USA) is inserted through the uterus 3 cm from the right lower edge of the uterine incision (or at this level if the patient was vaginally delivered) and 3 cm from the right lateral border without opening uterine cavity. The suture is threaded through the uterine cavity to pass from the anterior to the posterior aspects then passed over the fundus, and the uterus was repunctured 4-5 cm below the fundus and about 4 cm medial to the lateral border (Figure 1). The needle was then passed from the anterior to the posterior fundal region through the uterine cavity. The suture was again passed from the posterior aspect over the fundus to reach the anterior fundal region (Figure 2). The two ends of the thread were tied on the anterior wall as tightly as possible while an assistant applied bimanual compression. During such compression the vagina is checked to insure that the bleeding is controlled. The procedure was then repeated on the left (Figure 3). Care was taken not to damage the bowel and the bladder.

The patient's age, parity, gestation of pregnancy, presenting diagnosis, mode of delivery, and volume replacement were recorded (see Table 5). The age of patients was between 21 and 33 years (mean 27 years). Parity ranged from 0 to 3 as showed in Table 1, and the gestational age at which the procedure was performed ranged from 34 to 41 weeks (mean 38 weeks).

## 3. Results

Severe atonic PPH occurred in thirteen cases with risk factors for atony. The majority of patients (61.5%) were multiparas (see Table 1). Seven cases (53.8%) were referred to our units poorly managed, and urgent delivery was needed. The other six cases (46.2%) were admitted for antenatal care and managed in our unit (see Table 3). Caesarean section was done in ten cases (76.9%) and three cases (23.1%) delivered vaginally (as shown in Table 2). Compression suture was performed for major primary PPH caused by uterine atony in 10 cases after caesarean section and 3 cases after vaginal delivery. The uterine compression suture technique was sufficient to stop bleeding immediately in 92.2% of the women. In one woman (7.8%), the procedure failed, and hysterectomy was done (see Table 4). The total blood loss ranged from 2,000 to 6,000 mL (mean 4,000 mL). The hospital stay ranged from 4 to 7 days (mean 5 days). All the patients made a good postoperative recovery. None of the women developed complications related to the procedure. Only one patient had a secondary wound infection and was successfully managed. In all cases, followup (gynecological examination vaginal and abdominal ultrasound) done six weeks after delivery was normal. Hysteroscopic evaluation of uterine cavity for all successfully managed 12 cases was performed three months after the procedure and showed normal uterine cavities.

## 4. Discussion

PPH is a serious obstetric problem that threatens patient life. It is still one of the leading causes of maternal mortality and morbidity. Uterine atony is the most common cause of primary postpartum hemorrhage. Early diagnosis and management of risk factors greatly decrease its incidence [7]. In general, the management of atonic PPH includes patient resuscitations and simple medical measures escalating to invasive surgical procedures depending on patient response and hemodynamic state. Bilateral hypogastric artery ligation (BHAL) decreases bleeding by about 70%. However, it requires experience and surgical skill that is not available for many surgeons, and it is not without substantial risk of failure [8]. Major postpartum blood loss in hemodynamically unstable patients is more likely to need hysterectomy that can be one of the most dangerous procedures. Its complications include the permanent loss of fertility together with physical and psychological sequelae [9]. In 1997, a surgical technique for compression and apposition between the anterior and posterior walls of the uterus was described by Lynch in five women with postpartum hemorrhage [3]. Since its introduction into practice, the B-Lynch compression suture technique has been applied successfully worldwide and gained popularity [10], and variations on this compression technique have been described by other authors [4–6]. In our unit, a limited number of surgeons are familiar with internal artery ligation. So we try to practice compression suture technique for drug resistant-atonic PPH. B-Lynch suture was applied after the test of potential efficacy. However, we faced some difficulty due to slipping off suture at the lateral upper margins. In order to overcome this problem, we try to modify B-Lynch suture by firm transfixation of suture at uterine fundus. This was achieved by an additional firm puncture below the uterine fundus without obvious additional bleeding and tying the two ends anteriorly. In our study we faced 13 patients presented by severe atonic PPH. Initial general management was done and when failed, safety pin suture was applied after the test of potential efficacy (a simple bimanual compression after exteriorising the uterus) and was very effective in controlling bleeding with no need of additional surgical procedure in 12 cases (92.2%). Only in one case, the patient was referred to our unit presented by severe accidental hemorrhage and intrauterine fetal death. The patient was shocked with blood pressure 70/30 mmhg and feeble, rapid pulse (120 b/min.). Spontaneous vaginal delivery was achieved within 15 minutes with antishock measures done. Severe atonic PPH occurred with no response to massage and uterotonics drugs. Laparotomy was done with failure of safety pin suture to control bleeding. Hysterectomy was done, but unfortunately, DIC WAS superimposed, and the patient died after 4 hours. It is important to note that such suturing techniques may not achieve adequate control of bleeding particularly when there is coagulopathy and diffuse bleeding from an atonic uterus, and delay in effecting surgical technique may further compromise the patient. We have found that safety pin uterine compression suture is simple, effective, easy to learn, and can be easily performed during caesarean section as in vaginal delivery without risk of internal iliac artery ligation or hysterectomy complications. The time needed to complete the procedure decreased each time to be not more than 10 minutes by the end of the study and can be undertaken without opening the uterine cavity, which distinguishes it from the original B-Lynch suture with the advantage of decreasing the duration of anesthesia and blood loss. Also, many surgeons in our unit became familiar with the technique which helped in saving the life and future fertility of many patients. Complications of infections and intrauterine synechiae have been described after compression techniques [11]. In our series, none of the women developed complications related to the procedure, and follow-up diagnostic hysteroscopy done three month later for all successfully managed 12 cases showed clear uterine cavity with nice view of uterine ostia. Only in one case, gaped wound infection was successfully cleaned and repaired with secondary suture.

## 5. Conclusion

Safety pin uterine compression suture is a simple, safe, highly effective, and life-saving conservative procedure to control atonic postpartum hemorrhage with the advantage of preserving future fertility. All obstetric units which should have clear guidelines to deal with this emergency and techniques that allow preservation of the uterus should be applied when possible.

## Figures and Tables

**Table 1:** Distribution of parity of patients.

Parity	No. of patients	Percent
PG	5	38.5
P1	3	23.1
P2	3	23.1
P3	2	15.3
Total	13	100

**Table 2:** Mode of delivery.

Mode of delivery	No. of patients	Percent
Vaginal delivery	3	23.1
Elective CS	4	30.8
Emergency CS	6	46.1
Total	13	100

CS: Caesarean section.

**Table 3:** Place of managed labor.

Place of management	No. of patients	Percent
Referred	7	53.8
Inpatient	6	46.2
Total	13	100

**Table 4:** Outcome of compression suture.

Outcome of compression suture	No. of patients	Percent
Successful	12	92.2
Failure	1	7.8
Total	13	100

**Table 5:** Summary of characters of patients with severe atonic PPH treated by the use of safety pin uterine compression suture.

Age (yrs)	Parity	Gestation (wks)	Presenting diagnosis	Mode of delivery	Volume replacement
24	PG	39/40	Prolonged labor	Emergency LSCS	4 blood units
					2 FFP units
28	P1	36/40	Accidental hemorrhage	Vaginal delivery	8 blood units
					4 FFP units
21	PG	37/40	Severe PET	Emergency LSCS	4 blood units
23	PG	34/40	Severe PET,	Emergency LSCS	6 blood units
			HEELP syndrome		3 FFP units
28	PG	38/40	Twins pregnancy	Elective LSCS	4 blood units
					2 FFP units
27	PG	40/40	Obstructed labor	Emergency LSCS	7 blood units
					2 FFP units
33	P3	39/40	Prolonged labor	Vaginal delivery	10 blood units
					4 FFP units
29	P2	39/40	PGD, over sized baby	Elective LSCS	5 blood units
					2 FFP units
27	P2	37/40	Hydrocephalus, (PBD = 122 mm)	Elective LSCS	5 blood units
			polyhydramnios		2 FFP units
33	P3	38/40	Accidental hemorrhage,	Vaginal delivery	12 blood units
			(DIC)		6 FFP units
24	P1	41/40	Obstructed labor	Emergency LSCS	5 blood units
					2 units FFP
29	P2	38/40	Prolonged labor	Emergency LSCS	7 blood units
					3 FFP units
23	P1	36/40	Triplet pregnancy (ICSI)	Elective LSCS	5 blood units
					2 FFP units

LSCS: lower segment caesarean section. FFP: fresh frozen plasma. DIC: disseminated intravascular coagulation. PGD: pregestational diabetes. ICSI: intracytoplasmic sperm inoculation.
